# Eicosapentaenoic Acid Inhibits *KRAS* Mutant Pancreatic Cancer Cell Growth by Suppressing Hepassocin Expression and STAT3 Phosphorylation

**DOI:** 10.3390/biom11030370

**Published:** 2021-03-02

**Authors:** Ching-Feng Chiu, Ming-I Hsu, Hsiu-Yen Yeh, Ji Min Park, Yu-Shiuan Shen, Te-Hsuan Tung, Jun-Jie Huang, Hung-Tsung Wu, Shih-Yi Huang

**Affiliations:** 1 Graduate Institute of Metabolism and Obesity Sciences, Taipei Medical University, Taipei 11031, Taiwan; chiucf@tmu.edu.tw (C.-F.C.); hsumingi@yahoo.com.tw (M.-I.H.); da07107004@tmu.edu.tw (J.M.P.); 2 TMU Research Center of Cancer Translational Medicine, Taipei Medical University, Taipei 11031, Taiwan; 3 Nutrition Research Center, Taipei Medical University Hospital, Taipei 11031, Taiwan; 4 Department of Obstetrics and Gynecology, Wan Fang Hospital, Taipei Medical University, Taipei 11031, Taiwan; 5 School of Nutrition and Health Sciences, Taipei Medical University, Taipei 11031, Taiwan; ma07107015@tmu.edu.tw (H.-Y.Y.); DA07109003@tmu.edu.tw (Y.-S.S.); derossi83621@gmail.com (T.-H.T.); 6 Diet and Nutrition Department, Shuang Ho Hospital, Taipei Medical University, New Taipei 23561, Taiwan; 12255@s.tmu.edu.tw

**Keywords:** pancreatic cancer, *KRAS* mutation, hepassocin, eicosapentaenoic acid

## Abstract

Background: The oncogenic Kirsten rat sarcoma viral oncogene homolog (*KRAS*) mutation was reported to be the signature genetic event in most cases of pancreatic ductal adenocarcinoma (PDAC). Hepassocin (HPS/FGL1) is involved in regulating lipid metabolism and the progression of several cancer types; however, the underlying mechanism of HPS/FGL1 in the *KRAS* mutant PDAC cells undergoing eicosapentaenoic acid (EPA) treatment remains unclear. Methods: We measured HPS/FGL1 protein expressions in a human pancreatic ductal epithelial (HPNE) normal pancreas cell line, a *KRAS*-wild-type PDAC cell line (BxPC-3), and *KRAS*-mutant PDAC cell lines (PANC-1, MIA PaCa-2, and SUIT-2) by Western blot methods. HEK293T cells were transiently transfected with corresponding *KRAS*-expressing plasmids to examine the level of HPS expression with *KRAS* activation. We knocked-down HPS/FGL1 using lentiviral vectors in SUIT-2 cells and measured the cell viability by 3-(4,5-dimethylthiazol-2-yl)-2,5-diphenyltetrazolium bromide (MTT) and clonogenicity assays. Furthermore, a lipidomic analysis was performed to profile changes in lipid metabolism after HPS/FGL1 knockdown. Results: We found that the HPS/FGL1 level was significantly upregulated in *KRAS*-mutated PDAC cells and was involved in *KRAS*/phosphorylated (p)-signal transduction and activator of transcription 3 (STAT3) signaling, and the knockdown of HPS/FGL1 in SUIT-2 cells decreased cell proliferation through increasing G_2_/M cell cycle arrest and cyclin B1 expression. In addition, the knockdown of HPS/FGL1 in SUIT-2 cells significantly increased omega-3 polyunsaturated fatty acids (PUFAs) and EPA production but not docosahexaenoic acid (DHA). Moreover, EPA treatment in SUIT-2 cells reduced the expression of de novo lipogenic protein, acetyl coenzyme A carboxylase (ACC)-1, and decreased p-STAT3 and HPS/FGL1 expressions, resulting in the suppression of cell viability. Conclusions: Results of this study indicate that HPS is highly expressed by *KRAS*-mutated PDAC cells, and HPS/FGL1 plays a crucial role in altering lipid metabolism and increasing cell growth in pancreatic cancer. EPA supplements could potentially inhibit or reduce ACC-1-involved lipogenesis and HPS/FGL1-mediated cell survival in *KRAS*-mutated pancreatic cancer cells.

## 1. Introduction

Pancreatic cancer is the fourth leading cause of cancer-related deaths in the US and is the seventh leading cause of cancer deaths in Taiwan [1,2]. It was reported that the 5-year survival rate of pancreatic cancer only reaches about 9% in 2020 [1]. A lack of biomarkers for early pancreatic cancer detection and limited therapeutic options are leading causes of deaths in pancreatic cancer patients, which ultimately lead pancreatic cancer incidences to almost parallel its mortality.

The most commonly diagnosed type of pancreatic cancer is pancreatic ductal adenocarcinoma (PDAC), which arises from exocrine alteration of the pancreatic epithelium, and over 90% of PDAC patients exhibit a mutation of Kirsten rat sarcoma viral oncogene homolog (*KRAS*), which is a Ras family GTPase that activates phosphoinositide-3 kinase (PI3K) and mitogen-activated protein kinase (MAPK) pathways. Its mutation results in unstoppable cell division and ultimately leads to cancer formation [3]. In addition, it was reported that inflammatory signaling interacts with *KRAS-*regulated survival pathways and activates certain cytokines and the transcription factor, signal transducer and activator of transcription 3 (STAT3) [4]. Thus, if *KRAS* is mutated in pancreatic cancer cells, inflammatory signals cause further secretion of cytokines and also lead to dysregulated activation of STAT3 as positive feedback, fueling *KRAS*-driven pancreatic cancer [5]. Growing evidence indicates that mutant *KRAS* reprograms intracellular fatty acid (FA) metabolism to regulate lipid storage and utilization and promote cancer metastasis and progression [6,7,8]. Excess polyunsaturated fatty acids (PUFAs) administration can reduce the inflammatory response and therefore inhibit pancreatic cancer progression [9,10]. Previous studies pointed out that an intervention with n-3 PUFAs, such as eicosapentaenoic acid (EPA) and docosahexaenoic acid (DHA), promotes cell apoptosis to inhibit the growth rate of SW1990 pancreatic cancer cells [11]. Additionally, DHA and EPA decreased interleukin (IL)-6-induced C-reactive protein in HepG2 liver cancer cells by inhibiting STAT3 activity [12], which plays an important role in *KRAS*-induced pancreatic cancer growth and progression [13], suggesting that n-3 PUFAs can regulate intracellular inflammatory cytokines to influence *KRAS*-driven cell survival and the growth of pancreatic cancer.

Hepassocin (HPS), also known as fibrinogen-like protein 1 (FGL1), is expressed by the liver and weakly by the pancreas, and it exhibits mitogenic activity on isolated hepatocytes [14,15,16]. HPS was shown to mediate hepatic lipid accumulation to regulate hepatocyte proliferation and liver regeneration through activating the extracellular signal-regulated kinase 1/2 (ERK1/2) pathway [16,17,18]. Additionally, oleic acid, a steatogenic reagent, induces HPS expression in HepG2 cells, and knockdown of HPS decreases oleic acid-induced lipid accumulation [19], showing that HPS plays an important role in regulating hepatic lipid accumulation through upstream ERK1/2 signaling. Another study also suggested that the binding site of STAT3 and HNF1 is found in the promoter region of HPS and contributes to the transcriptional regulation of HPS [18]. Previous studies suggested that the low expression of HPS was also associated with liver cancer progression, because the downregulation of HPS in hepatocellular carcinoma (HCC) cells led to an increase in cell proliferation and colony-forming capacity in vitro [18]. In gastric cancer (GC), HPS was reported to be upregulated in GC tissues compared to normal tissues, and high HPS was directly correlated with poorer prognoses of GC patients [20]. Although several studies found an association between HPS and cancer progression, the role and functions of HPS in pancreatic cancer cells have yet to be elucidated. Thus, in this study, we investigated both the expression and the role of HPS in KRAS-mutated PDAC cell lines, and we analyzed lipidomic differences, including n-3 and n-6 PUFAs, in HPS-knockdown pancreatic cancer cells. Intriguingly, we found that EPA treatment dominantly inhibit the cell survival of SUIT-2 KRAS-mutated PDAC and ultimately reduce STAT3 phosphorylation and HPS expression.

## 2. Materials and Methods

### 2.1. Reagents

All cell culture-related media were purchased from GE Healthcare Life Sciences (Thermo Fisher Scientific, Waltham, MA, USA). Fetal bovine serum (FBS) was purchased from Corning^®^ (Corning, NY, USA). A penicillin–streptomycin solution was purchased from TOKU-E (Bellingham, WA, USA). The n-3 PUFAs we used were α-linolenic acid (ALA, *cis*,*cis*,*cis*-9,12,15-octadecatrienoic acid, L2376, Sigma-Aldrich, St. Louis, MO, USA), EPA (*cis*-5,8,11,14,17-eicosapentaenoic acid, E2011, Sigma-Aldrich), and DHA (*cis*-4,7,10,13,16,19-docosahexaenoic acid, D2534, Sigma-Aldrich), and the n-6 PUFAs we used were linoleic acid (LA, 9-*cis*,12-*cis*-linoleic acid, L1376, Sigma-Aldrich) and arachidonic acid (AA, *cis,cis,cis,cis*-5,8,11,14-eicosatetraenoic acid, A3611, Sigma-Aldrich). Polyethyleneimine (PEI) and polybrene were purchased from Millipore (Darmstadt, Germany). A protease inhibitor cocktail was purchased from Roche (Basel, Switzerland).

### 2.2. Cell Culture

Nonmalignant HPNE and PDAC cell lines (BxPC-3, PANC-1, MIA Paca-2, and SUIT-2) were obtained from Dr. Wun-Shaing Wayne Chang and Dr. Chen, Li-Tzong, National Institute of Cancer Research (National Health Research Institutes, Miaoli, Taiwan). Human embryonic kidney (HEK293T) cells were purchased from the Bioresource Collection and Research Center (BCRC, Hsinchu, Taiwan) and grown in culture medium according to BCRC instructions. These cells were free of mycoplasma contamination, and their identity was confirmed by short tandem repeat (STR) profiling at the Center for Genomic Medicine (National Cheng-Kung University, Tainan, Taiwan) and BCRC.

### 2.3. Ras Activation

HEK293T cells were seeded in six-well plates overnight and then transfected with 2 μg of pUSE-Ampicilin (vector), pCMV-*RAS*-wild-type (*RAS*-WT), *RAS*-constitutively active (*RAS*-V12), or *RAS*-dominant-negative (*RAS*-N17) plasmids (Clontech, Palo Alto, CA, USA) with 0.2 μg of the pAS2.EGFP.puro plasmid (GFP; RNAi core, Academia Sinica, Taiwan) using the DreamFect™ Gold transfection reagent (OZ Biosciences, Marseille, France). After 2 days of incubation, cells were starved for 6 h in serum-free Dulbecco’s modified Eagle medium (DMEM) and collected for a Western blot analysis.

### 2.4. Lentiviral Production and Infection

Short hairpin (sh)RNAs carrying a puromycin selection marker were purchased from the National RNAi Core Facility of Academic Sinica (Taipei, Taiwan). For lentiviral production, pCMV deltaR8.91, and pCMV-VSV-G were co-transfected into HEK293T cells, and the transduction media was collected. Then, SUIT-2 cells were transfected with three shHPS plasmids of shHPS#20, 5′-GCTAGTCACCAAAGAATGAAA-3′; shHPS#21, 5′-CTGAACATATCCATGCGCAAT-3′; shHPS#90, 5′-GAAGTCCAGTTCCTTGATAAA-3′, and control plasmids of shScramble, TRC2.Scramble; shVoid, TRC2.Void; and shLKO, TRC025, using PEI transfection. Cells were treated with 2 μg/mL puromycin to select successfully transfected stable cells, which were pooled for subsequent analyses.

### 2.5. Western Blotting

Following the knockdown of HPS (shHPS#20, shHPS#21, and shHPS#90) and controls (Scramble, Void, and LKO), SUIT-2 cells were lysed in RIPA buffer containing a protease inhibitor cocktail and phosphatase inhibitor cocktail (Roche, Boston, MA, USA). Equal amounts of total protein were resolved by sodium dodecylsulfate polyacrylamide gel electrophoresis (SDS-PAGE) and transferred to polyvinylidene difluoride membranes. Membranes were blocked with 5% skim milk in TBST (Tris-buffered saline containing 0.1% Tween-20) for 30 min and then incubated overnight at 4 °C with the indicated primary antibodies: β-actin (Millipore, Billerica, MA, USA; 1:7500), α-tubulin (ABclonal, Cambridge, MA, USA; 1:7500), GAPDH (GeneTex, Irvine, CA, USA; 1:7500), HPS (FGL1; Proteintech Group, Chicago, IL, USA; 1:1000), STAT3 (Cell Signaling, Beverly, MA, USA; 1:5000), phosphorylated (p)-STAT3 (Tyr705, D3A7, Cell Signaling; 1:5000), cyclin B1 (Cell Signaling; 1:1000), cyclin D1 (Cell Signaling; 1:1000), cyclin E (Cell Signaling; 1:1000), acetyl coenzyme A carboxylase (ACC)-1 (Cell Signaling; 1:1000), and FA synthase (FAS; Cell Signaling; 1:1000). Membranes were washed with TBST and incubated for 1 h at room temperature with the appropriate secondary antibodies conjugated to horseradish peroxidase. Then, membranes were washed, signals from the immunoreactive bands were detected using an electrochemiluminescence reagent (WBLUF0500; Burlington, MA, USA), and resulting bands were achieved using the UVP biochemical system and VisionWorks LS software (VisionWorks, Cedar Rapids, IA, USA).

### 2.6. Cell Viability Assay

Approximately 5 × 10^3^ cells were seeded per well in 96-well plates. After 24, 48, 72, and 96 h of incubation, 25 μL of a 3-(4,5-dimethylthiazol-2-yl)-2,5-diphenyltetrazolium bromide (MTT) solution (5 mg/mL in phosphate-buffered saline (PBS)) was added to each well, and cells were incubated for another 4 h at 37 °C. Then, the medium was completely discarded, and 100 μL of dimethyl sulfoxide (DMSO) was added for 10 min at room temperature to dissolve the MTT formazan crystals. The absorbance was measured with an EPOCH2 microplate reader (BioTek, Winooski, VT, USA) at a wavelength of 570 nm.

For clonogenicity assays, cells were plated into six-well cell culture plates at a density of 100 cells/well. Media were replaced with fresh medium every 3 days, and cells were allowed to grow for 3 weeks. At the end of the experiment, the growth medium was removed, and cells were washed twice with ice-cold PBS. Then, colonies were fixed with 10% formalin for 30 min and stained with 0.5% crystal violet in 2% methanol for 1 h for enumeration. Photos were taken with a digital camera, and the number of cell colonies was counted with VisionWorks software.

### 2.7. Cell Cycle Analysis

Cell suspensions were centrifuged at 1500 rpm at room temperature for 10 min. Supernatants were removed, washed with cold PBS, and fixed with 66% cold ethanol (EtOH) at 4 °C for 2 h. Cells were then re-washed with PBS, and a staining mixture was added that contained 0.2 mg/mL propidium iodide (PI) and 20 μg/mL RNase A. After being re-suspended, cells were incubated at room temperature for 30 min and analyzed by flow cytometry (Invitrogen AttuneNxt) using Flowjo software (Becton, Dickinson & Company, Franklin Lakes, NJ, USA).

### 2.8. Lipidomic Analysis

To determine the global lipid metabolic profiles, 5 × 10^6^ of HPS-knocked-down and HPS-control SUIT-2 cells were added to a 2-fold volume of lysis buffer with an ultrasonic processor (amplitude: 20%, pulse on/off: 3 s/1 s, time: 12 s); then, 1.5 mL of methanol was added to the collected cell lysate and mixed well, and then, 3 mL of chloroform was added with vortexing and shaken at room temperature for 1 h. To the total extract, 1.25 mL H_2_O was added, mixed well, and centrifuged at 3000 rpm and 4 °C for 10 min. After centrifugation, the collected bottom layer was dried with a vacuum pump and stored at −80 °C. For liquid chromatographic tandem mass spectroscopic (LC-MS/MS) analyses, all extracts were re-dissolved in an isopropanol/acetonitrile/water (2:1:1) solution and analyzed on an ACQUITY UPLC CSH C18 column with a SYNAPT G2 HDMS mass spectrometer (Waters, Milford, MA, USA, by the Mass Spectrum Core Facility, Taipei Medical University). The Alliance LC system and quadrupole time of flight (Q-TOF) micro™ instrument were controlled using MassLynx^®^ software vers. 4.0 (Waters). Automated processing of the acquired mass spectra, identification, and quantification of detected lipid species, such as monoglycerides (MGs), diglycerides (DGs), triglycerides (TGs), phosphatidic acid (PA), phosphatidylethanolamine (PE), phosphatidylglycerol (PG), phosphatidylinositol (PI), phosphatidylserine (PS), sphingomyelin (SM), ceramide (Cer), ALA, EPA, DHA, cis-9, cis-12-octadecadienoic acid (LA), and AA were analyzed by Progenesis QI^TM^ software (Nonlinear Dynamics, Newcastle, UK).

### 2.9. Statistical Analysis

All statistical analyses were performed with Prism 8 software (La Jolla, CA, USA). Data are presented as the mean ± standard deviation (SD), and statistical significance was assessed by a one-way analysis of variance (ANOVA) followed by a two-sided Tukey’s test. A value of *p* < 0.05 was considered statistically significant.

## 3. Results

### 3.1. Expression of HPS in KRAS-Mutant Pancreatic Cancer Cells

**Figure 1:**
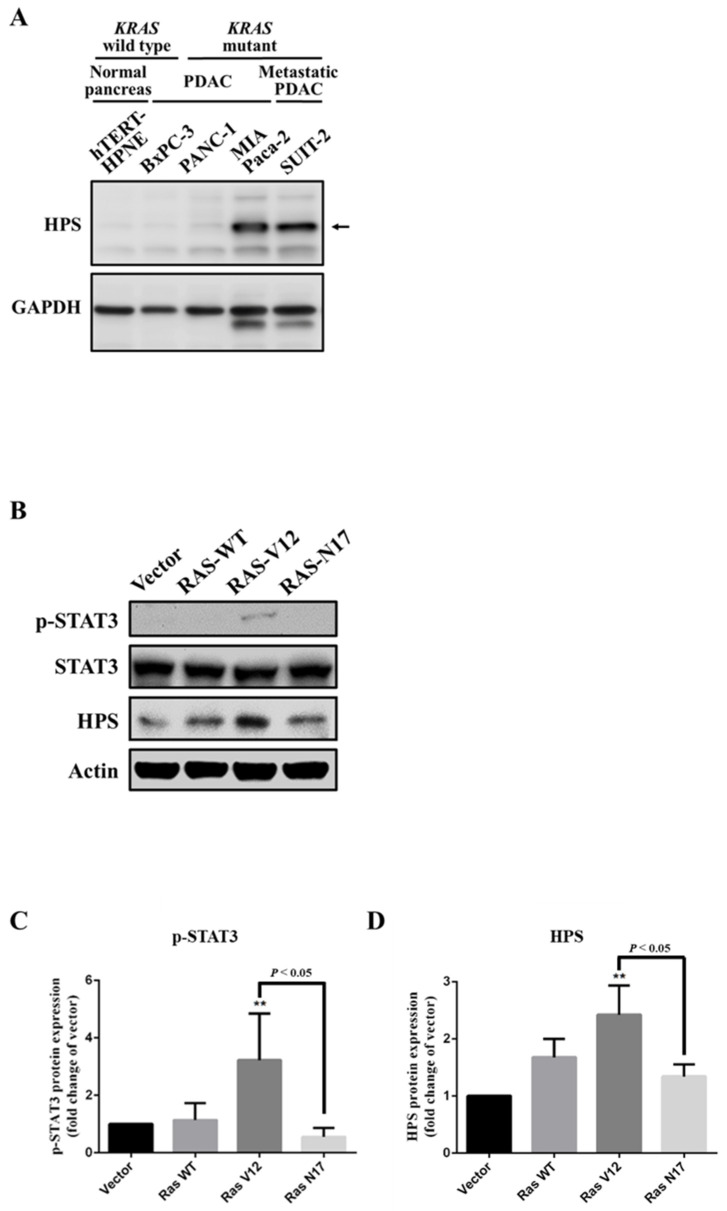
Expression level of hepassocin (HPS) in Kirsten rat sarcoma viral oncogene homolog *(KRAS)*-mutant pancreatic cancer cells. (**A**) Protein expressions of HPS and glyceraldehyde-3-phosphate dehydrogenase (GAPDH) by hTERT-HPNE, BxPC-3, PANC-1, MIA Paca-2, and SUIT-2 cells were measured by a Western blot analysis. GAPDH was used as the internal control. (**B**) Protein expressions of phosphorylated (p)-STAT3, STAT3, and HPS by HEK293T cells transiently transfected with the *RAS* wild-type (WT), constitutively active *RAS* (V12), and dominant negative *RAS* (N17) plasmids and a control vector were measured by a Western blot analysis. Actin was used as the internal control. The p-STAT3/STAT3 ratio (**C**) and HPS (**D**) expression level of indicated cells were analyzed with ImageJ software, normalized to actin, and evaluated as multiples of change compared to vector-transfected cells. Results are shown as the mean ± SD of three independent experiments. ** *p* < 0.01 by a one-way ANOVA followed by Tukey’s post-hoc test.

To examine the importance of HPS expression in *KRAS*-mutant PDAC cells, we analyzed HPS protein levels in *KRAS* wild-type (WT) and mutant PDAC cell lines (BxPC-3, PANC-1, MIA Paca-2, and SUIT-2) and a normal epithelial pancreatic cell line (hTERT-HPNE). Results revealed that HPS protein expression was significantly lower in hTERT-HPNE, *KRAS* wild-type, and BxPC-3 cells, compared to those in the *KRAS*-mutant PDAC cell lines of PANC-1 and MIA Paca-2, and metastatic SUIT-2 cells, illustrating a trend of higher expression of HPS in most *KRAS*-mutant PDAC cell lines (Figure 1A). We next compared HPS expressions in HEK293T cell lines carrying the *RAS*-WT, *RAS*-constitutively active (*RAS*-V12), and *RAS*-dominant negative (*RAS*-N17) mutations. We found that in transfected *KRAS*-mutant *RAS*-V12 cells, p-STAT3 was activated (Figure 1B,C) and HPS expression was significantly higher (Figure 1B,D) compared to *RAS*-WT and *RAS*-N17-transfected cells, suggesting that active KRAS/STAT3 signaling might be involved in regulating HPS expression and HPS-mediated biofunctions in PDAC cells.

### 3.2. Knockdown of HPS in SUIT-2 Cells Decreased Cell Growth and Induced Cell Cycle Arrest

**Figure 2:**
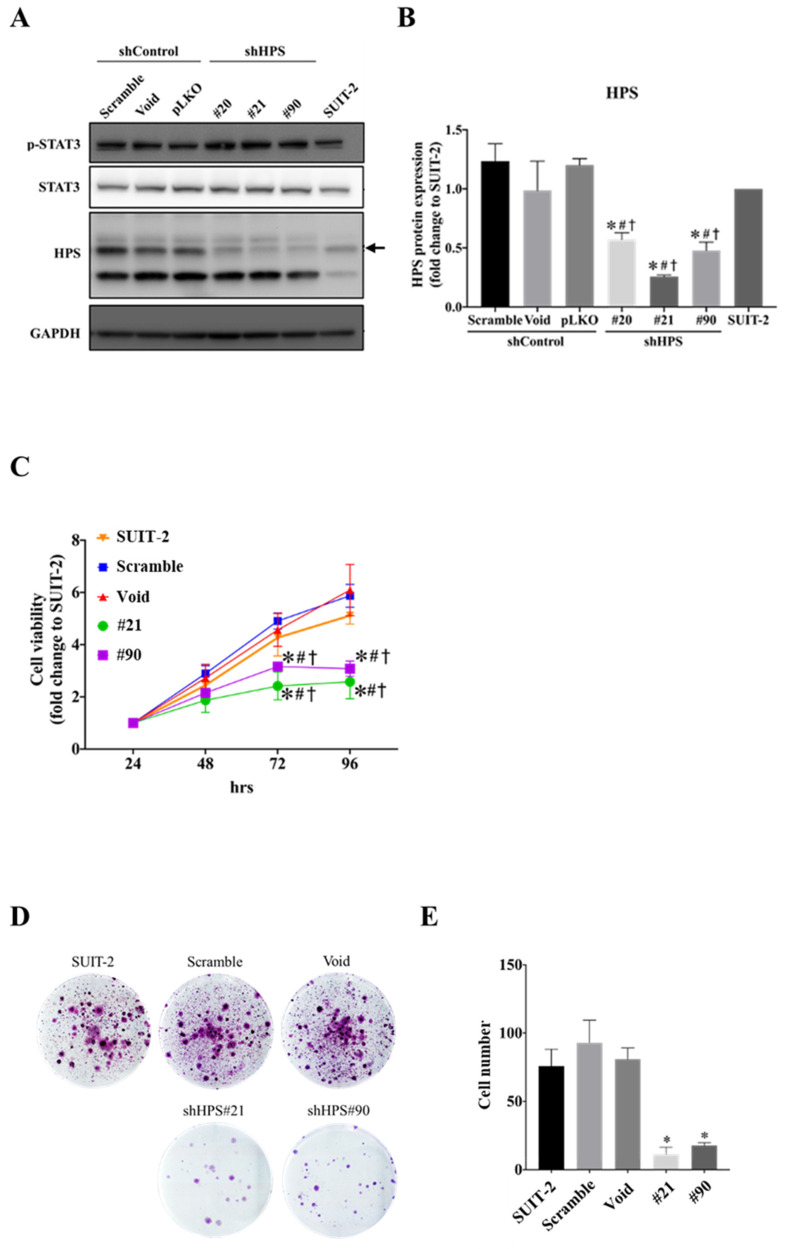
Knockdown of hepassocin (HPS) decreases pancreatic cancer cell growth in SUIT-2 cells. (**A**) SUIT-2 cells were infected with a lentivirus carrying the short hairpin (sh) control vectors of Scramble, Void, and pLKO, and shHPS vectors #20, #21, and #90, and protein expressions of STAT3, phosphorylated (p)-STAT3, HPS, and GAPDH of stably transfected SUIT-2 cells were measured by a Western blot analysis. GAPDH was used as the internal control. (**B**) HPS expression levels of the indicated SUIT-2 cells were analyzed with ImageJ software, normalized to GAPDH, and evaluated as multiples of change compared to SUIT-2 cells. Results are shown as the mean ± SD of three independent experiments. * *p* < 0.05 vs. Scramble, ^#^ *p* < 0.05 vs. Void, and ^†^ *p* < 0.05 vs. pLKO control (by a one-way ANOVA followed by Tukey’s post-hoc test). (**C**) The indicated SUIT-2 cells were seeded and cultured for a period of time (24, 48, 72, and 96 h), and cell viability was subsequently evaluated by an MTT assay. Multiple changes of cell viability were compared to each 24-h group. Results are shown as the mean ± SD of three independent experiment, each performed in triplicate. * *p* < 0.05 vs. Scramble, ^#^ *p* < 0.05 vs. Void, and ^†^ *p* < 0.05 vs. pLKO control (by a one-way ANOVA followed by Tukey’s post-hoc test). (**D**) The cell clonogenicity effect of HPS knockdown on SUIT-2 cells was measured by a clonogenic assay. (**E**) Total cell numbers were measured as the number of colonies Size (pixel*2) = 100-infinity calculated by ImageJ. * *p* < 0.05 vs. the Scramble control.

To understand the role and function of HPS in pancreatic cancer cells, we knocked down HPS expression in SUIT-2 pancreatic cancer cells using lentiviruses. After a week of puromycin selection, SUIT-2 cells with stable knockdown of HPS (shHPS#20, shHPS#21, and shHPS#90) were collected, and the decreased HPS level in SUIT-2 cells was confirmed by Western blotting when compared to the shControl groups (Scramble, Void, and pLKO) (Figure 2A,B; *p* < 0.05). Additionally, we found that knockdown of HPS did not alter both phosphorylation and total levels of STAT3 (Figure 2A), whereas we have shown previously that active KRAS/p-STAT3 increased HPS expression (Figure 1B–D), which is in agreement with the previous study that found that STAT3 and HNF1 bind to the HPS promoter and transcriptionally downregulate HPS in HCC [18]. Therefore, our result implies that KRAS/p-STAT3 mediates the regulation of HPS expression in SUIT-2 pancreatic cancer cells.

**Figure 3:**
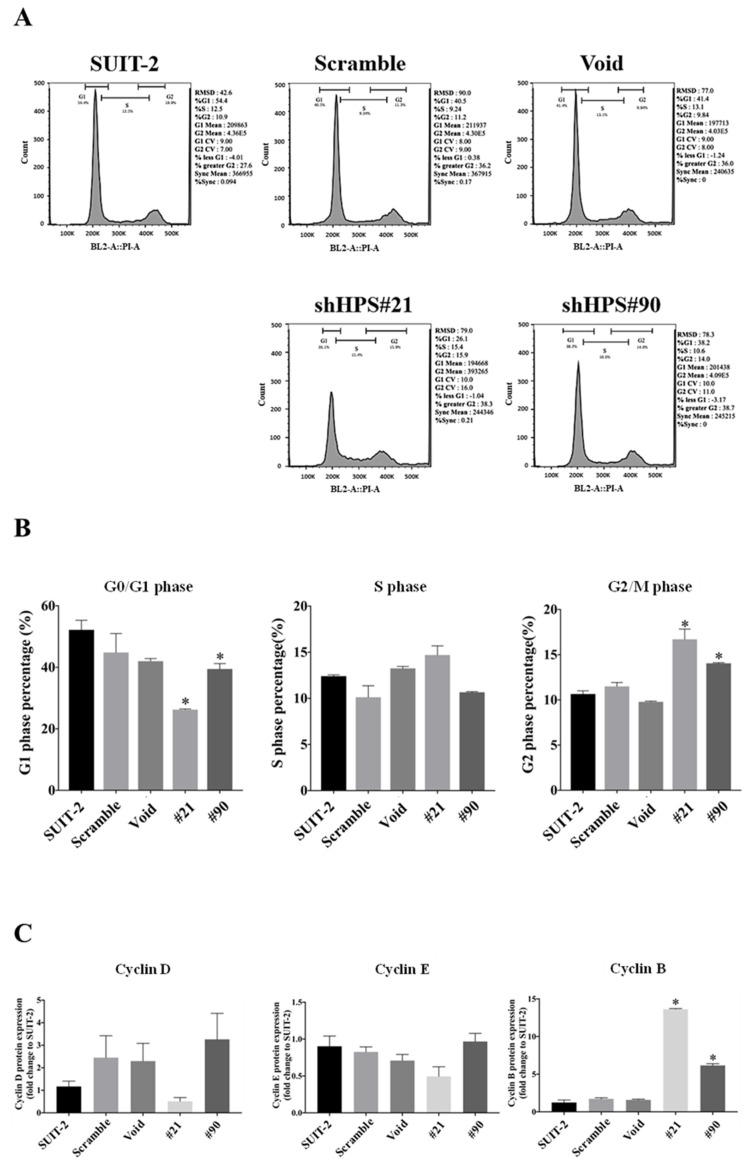
Knockdown of hepassocin (HPS) induces cell cycle arrest in SUIT-2 cells. (**A**) SUIT-2 cells were infected with a lentivirus carrying control vectors of Scramble and Void and the short hairpin (sh) HPS vectors #21 and #90, the indicated cell pellets were fixed with 60% EtOH at 4 °C and stained with propidium iodide and RNase A, and their DNA contents were analyzed by flow cytometry. (**B**) G_0_/G_1_, S, and G_2_/M phase percentages of the indicated SUIT-2 cells were determined by FlowJo software using the Dean–Jett–Fox model (w/sync. peak). (**C**) Cyclin D, B, and E expression levels of the indicated SUIT-2 cells were analyzed by Western blotting and quantified with ImageJ software, normalized to tubulin, and evaluated as multiples of change compared to SUIT-2 cells. Results are shown as the mean ± SD of three independent experiments. * *p* < 0.05 vs. SUIT-2 (by a one-way ANOVA followed by Tukey’s post-hoc test).

We also examined the cell cycle distribution of SUIT-2 cells with HPS knockdown by flow cytometry, as shown in Figure 3A,B. SUIT-2 cells with HPS knockdown exhibited a significantly decreased G_0_/G_1_ phase and increased G_2_/M phase compared to the controls. Moreover, cell cycle-associated proteins, such as cyclin D, cyclin E, and cyclin B1, were analyzed by Western blotting (Figure S2), and the results showed a significantly increased cyclin B1 level in SUIT-2 cells with HPS knockdown (Figure 3C), suggesting that HPS knockdown accumulated cyclin B1 expression and induced G_2_/M cell cycle arrest.

### 3.3. Effects of HPS on Lipid Contents and Composition in SUIT-2 Cells

To further explore the effects of HPS on pancreatic cancer cells, we extracted lipid contents and analyzed the lipidomic composition between the controls (Void and Scramble) and HPS knockdown (shHPS#21 and shHPS#90) SUIT-2 cells by LS/MS/MS with a principal component analysis (PCA) and orthogonal partial least squares discriminant analysis (OPLS-DA). As shown in Supplementary Figure S1A, both the PCA score plot and OPLS-DA plot showed a substantial difference between control and HPS-knockdown SUIT-2 pancreatic cancer cells. Results revealed that abundances of C16:0, C24:6, and C18:0 MGs were significantly reduced in HPS-knockdown SUIT-2 cells (Supplementary Figure S1B, left panel). Abundances of C11:3/11:3, 16:1/24:4, and C22:3/22:4 DGs were also significantly lower in HPS-knockdown cells than in control cells, while only HPS-knockdown (shHPS#90) SUIT-2 cells showed a significantly lower abundance of 22:4/24:4 and 22:5/22:5 compared to the controls (Supplementary Figure S1B, middle panel). On the other hand, abundances of C44:0, C46:1, C46:2, C48:1, C49:3, C50:0, C50:1. C52:2, C52:3, C53:1, C53:3, C54:5, C55:5, C56:2, C56:3, C58:4, C58:8, C60:8, and C62:12 TGs were significantly higher in HPS-knockdown cells than in control cells (Supplementary Figure S1B, right panel). Levels of phospholipids, including PA, PC, and PE, were significantly reduced in HPS-knockdown cells, and total levels of PI, PS, and Cer remained largely unchanged (Supplementary Figure S1C).

**Figure 4:**
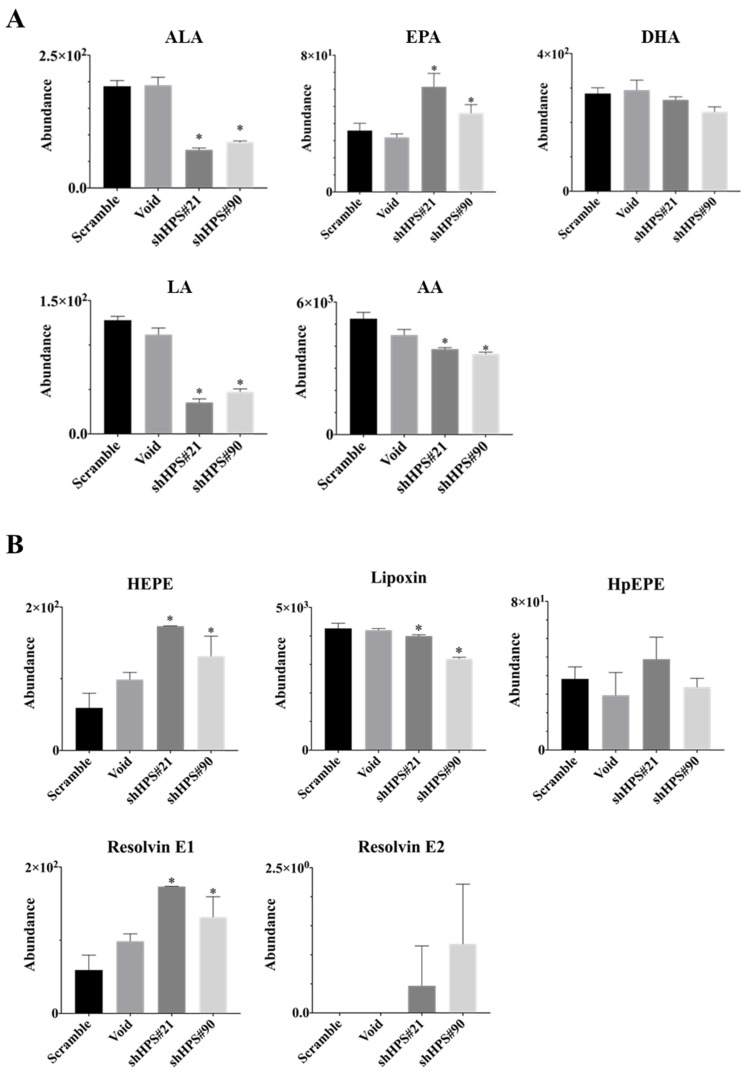
Knockdown of hepassocin (HPS) in SUIT-2 cells changes the lipid profile. (**A**) Expression levels of α-linolenic acid (ALA), eicosapentaenoic acid (EPA), docosahexaenoic acid (DHA), linoleic acid (LA), and arachidonic acid (AA). (**B**) Abundances of hydroxyeicosapentaenoic acid (HEPE), lipoxin, hydroperoxyeicosapentaenoic acid (HpEPE), Resolvin E1, and Resolvin E2 in SUIT-2 cells with knockdown of HPS. Control vectors of Scramble and Void, and short hairpin (sh) HPS vectors #21 and #90. Results are shown as the mean ± SD of three independent experiments. * *p* < 0.05 vs. the control (by a one-way ANOVA followed by Tukey’s post-hoc test).

Levels of n-3 and n-6 PUFAs were also identified and showed significantly decreased levels of an n-3 PUFA (ALA), n-6 PUFAs (LA and AA), AA-mediated hydroperoxyeicosapentaenoic acid (HpEPE), and lipoxin, but there was no difference in DHA in SUIT-2 cells with HPS knockdown (Figure 4A,B). Notably, significantly higher levels of EPA, EPA-mediated hydroxyeicosapentaenoic acid (HEPE), resolvin E1, and resolvin E2 production were recorded in SUIT-2 cells with HPS knockdown (Figure 4A,B), implying that HPS expression is involved in regulating n-3 and n-6 PUFA metabolism and pancreatic cancer cell growth. Taken together, EPA and its downstream products could potentially be used to inhibit HPS-mediated cell survival and the growth of pancreatic cancer.

### 3.4. EPA Decreases Cell Survival and HPS Expression of SUIT-2 Cells

**Figure 5:**
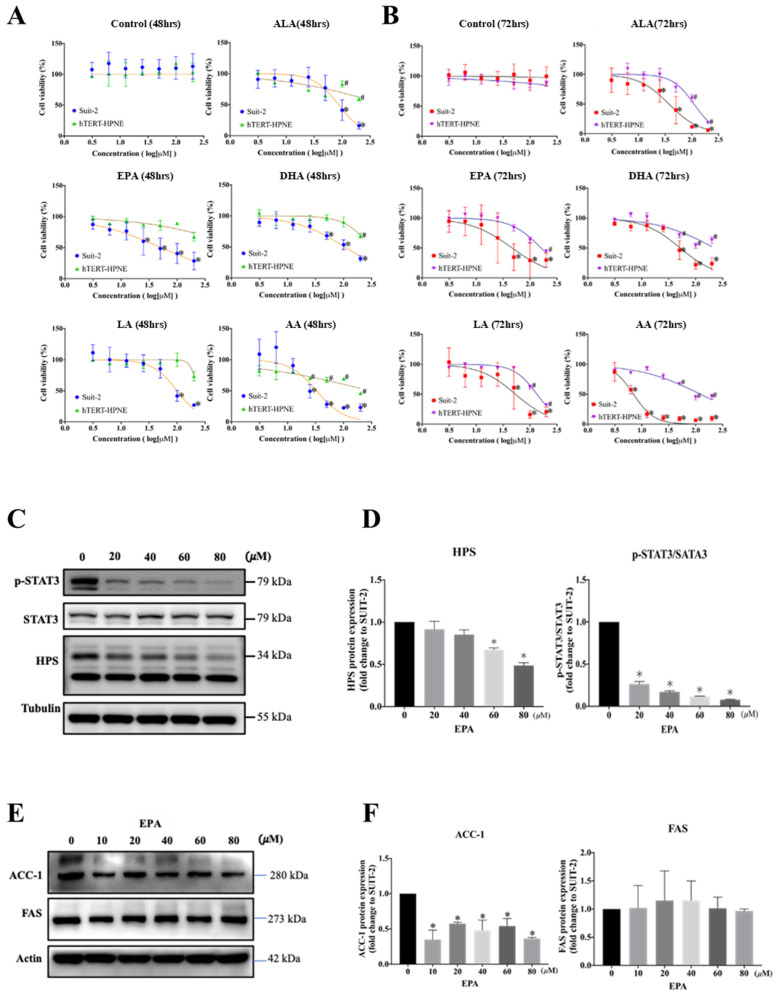
Treatment with n-3 and n-6 polyunsaturated fatty acids (PUFAs) decreases the viability of SUIT-2 cells. SUIT-2 and hTERT-HPNE cells were exposed to different concentrations (0, 3.125, 6.25, 12.5, 25, 50, 100, and 200 μM) of n-3 or n-6 PUFAs for 48 (**A**) and 72 h (**B**), and cell viability was subsequently evaluated by a 3-(4,5-dimethylthiazol-2-yl)-2,5-diphenyltetrazolium bromide (MTT) assay. Ethanol (75%) was used to prepare the solution and as serial dilution controls. The percentage of cell viability is shown relative to the untreated controls. Results are shown as the mean ± SD of three independent experiments, each performed in triplicate. * *p* < 0.05 vs. untreated SUIT-2 cells, ^#^ *p* < 0.05 vs. untreated hTERT-HPNE cells (by a two-way ANOVA followed by Tukey’s post-hoc test). (**C**) Protein expressions of signal transduction and activator of transcription 3 (STAT3), phosphorylated (p)-STAT3, and HPS of the indicated SUIT-2 cells were measured by a Western blot analysis. Tubulin was used as the internal control. (**D**) Expression levels of p-STAT3/STAT3 and HPS were analyzed with ImageJ software, normalized to actin, and evaluated as multiples of change compared to untreated cells. Results are shown as the mean ± SD of three independent experiments. * *p* < 0.05 (by a one-way ANOVA followed by Tukey’s post-hoc test). (**E**) Protein expressions of ACC-1 and FAS of the indicated SUIT-2 cells were measured by a Western blot analysis. Actin was used as the internal control. (**F**) Expression levels of ACC-1 and FAS were analyzed with ImageJ software, normalized to actin, and evaluated as multiples of change compared to untreated cells. Results are shown as the mean ± SD of three independent experiments. * *p* < 0.05 (by a one-way ANOVA followed by Tukey’s post-hoc test).

To further investigate whether n-3 and n-6 PUFA treatment of pancreatic cancer cells could be involved in HPS expression and cell survival, we treated cells with various dosages (0, 25, 50, 100, and 200 μM) of either n-3 PUFAs (ALA, EPA, and DHA) or n-6 PUFAs (LA and AA) for 48 and 72 h and measured the SUIT-2 pancreatic cancer cell viability using an MTT assay. Nonmalignant pancreas epithelial hTERT-HPNE cells were used to test the toxicity compared to SUIT-2 cells. As shown in Figure 5A,B, ALA, EPA, DHA, LA, and AA treatments significantly reduced the cell viability of SUIT-2 cells compared to hTERT–HPNE cells at 48 and 72 h. Notably, EPA, LA, and AA suppressed the cell survival of SUIT-2 cells at a higher concentration for 48 h but not hTERT-HPNE cells (Figure 5A). We also analyzed the half maximal inhibitory concentration (IC_50_) of different PUFAs between hTERT-HPNE cells and SUIT-2 cells using a nonlinear regression method. IC_50_ values of ALA, EPA, DHA, LA, and AA treatments in SUIT-2 cells were 88.46, 51.75, 104.4, 99.81, and 32.79 μM at 48 h and were 37.17, 46.48, 51.7, 53.74, and 7.014 μM at 72 h, respectively. IC_50_ values of ALA, EPA, DHA, LA, and AA treatments in hTERT-HPNE cells were 571, 1354, 307.2, 230.3, and 292 μM at 48 h and 109.4, 160.8, 221.9, 134.4, and 122.3 μM at 72 h, respectively (Table 1). Results showed that EPA-treated SUIT cells had lower IC_50_ levels than did hTERT-HPNE cells.

We next examined whether EPA treatment affected p-STAT3 and HPS expressions by SUIT-2 cells and found that EPA significantly reduced the p-STAT3/STAT3 expression ratio and HPS protein levels at concentrations of 60 and 80 μM in SUIT-2 cells at 48 h (Figure 5C,D), indicating that EPA might reduce *KRAS*/p-STAT3-mediated HPS expression and cell growth in pancreatic cancer cells. A previous study showed that EPA suppressed ACC activity, which plays an important role in FA metabolism; thus, we next examined protein levels of ACC-1 and long-chain FA synthases (FAS and FASN) to determine their involvement in EPA-mediated inhibition of SUIT-2 pancreatic cancer cell viability. Results revealed that the protein expression of ACC-1 was significantly reduced with 10, 20, 40, 60, and 80 μM of EPA treatments, whereas FAS protein expression remained largely unchanged with various doses of EPA treatment (Figure 5E,F). Overall, these data reveal that EPA reduces p-STAT3/HPS expression and ACC-1-mediated FA metabolism to inhibit the viability of SUIT-2 pancreatic cancer cells.

## 4. Discussion

Polyunsaturated fatty acids (PUFAs) are units of the cell membrane, including n-3 and n-6 PUFAs. Recent studies have shown that n-3 PUFAs play a vital role in cell signal transduction, cell structure, and the mobility of the membrane [21], and they raise the response rate to chemotherapy in cancer patients [22]. These changes in membrane compositions affect receptor activity, signal molecule production, and lipid mediator production, to arouse variation in metabolism causing the cells and tissue level [23]. n-3 PUFA and its mediators have anti-inflammatory and anti-nociceptive effects due to inhibiting angiogenesis, inflammation and cancer growth, including reducing the release of arachidonic acid from the cell membranes [24]. Several studies reported that the potential capabilities of n-3 PUFAs, including DHA and EPA, not only improve the efficacy and tolerability of conventional anti-cancer treatments but also protect the host from drug-related toxicity [25,26]. Additionally, dietary n-3 PUFAs in genetic *KRas^G12D^* mutant mice in vivo and in vitro show that n-3 PUFAs physically merged into a phospholipids layer of the cellular membrane to reduce the lateral segmentation of cholesterol-dependent and independent nanoclusters and accordingly inhibit the interaction of oncogenic *KRAS* signaling effectors [27], implying that the disruption of membrane nanoclustering might overcome oncogenic *KRAS*-induced tumorigenesis and cancer progression. Indeed, n-3 PUFAs induce apoptosis and suppress cell proliferation in *KRAS*-mutant-derived pancreas nestin-expressing HPNE-*KRAS^G12D^* cells both in vitro and in vivo by reducing AKT phosphorylation [28]. A recent paper reported that DHA promotes the cell apoptosis of PANC-1 pancreatic cancer cells by inducing DNA fragmentation, activating caspase-3, and increasing the ratio of Bax/Bcl-2 via downregulating STAT3/nuclear factor (NF)-κB/cyclin D1 signaling [29]. On the other hand, EPA treatment of adipocytes reduces adipocyte-secreted factors, thus inhibiting breast cancer cell inflammation and migration [30]. Morevover, EPA attenuates obesity-related hepatocellular carcinogenesis development through inhibiting obesity-induced STAT3 activation [31]. A high expression of phosphorylated STAT3 was shown in liver metastatic pancreatic (LMP) cell lines derived from *LSL-Kras^G12D/+^/Pdx1^Cre/+^* (KC) mice, compared to pancreatic intraepithelial neoplasia (PanIN) or primary PDAC cells [32], suggesting that STAT3 activation is an important factor that promotes metastatic and advanced stages of pancreatic cancer. Consistent with those studies, our study demonstrated that n-3 PUFA treatments, especially with EPA, significantly reduced cell viability and p-STAT3 expression of liver metastatic pancreatic cancer SUIT-2 cells compared to that of normal epithelial pancreas hTERT-HPNE cells. 

Cancer cells regulate lipolysis and lipogenesis processes to promote rapid cell growth and invasive progression. The uptake and synthesis of free fatty acids (FFAs) constitute metabolic reprogramming in cancer cells to sustain cell proliferation and impact cell migration by altering membrane fluidity [33]. For example, FFAs treatment increases the expression of plasminogen activator inhibitor-1 and SMAD4 to promote cell invasion and metastasis in breast cancer cells [34]. For fatty acids synthesis, ATP-citrate lyase (ACLY) catalyzes the conversion of citrate and coenzyme A (CoA) to acetyl-CoA, and the rate-limiting enzyme, acetyl-CoA carboxylase (ACC), converts acetyl-CoA into malonyl-CoA, which is the substrate for FAs synthesis and is involved in the elongation of FAs through fatty acid synthase (FAS). *KRAS* activates lipogenesis, which is related to the specific induction of FAS, and this activation leads to different proteomics and lipid signatures in lung cancer cells [35]. A high expression of FAS has been reported in many cancers, including colorectal cancer, lung cancer, hepatocellular carcinoma, pancreatic cancer, and gastric cancer [36]. However, it has been shown that FAS inhibitors, such as orlistat and cerulenin, have produced severe side effects in mice with tumor model [37,38], and stable FAS-silencing lung cancer A549 cells has been found to unexpectedly increase cell migration and lung metastasis in vivo [39]. Accumulating studies reported that ACC-1 is overexpressed in human cancer cells and is involved in de novo lipogenesis and the development of tumors. The knockdown of ACC-1 with small interfering (si)RNA induced significant cell apoptosis in prostate cancer cells and cell migration in hepatoma HepG2 cells [40,41], and dual ACC-1/ACC-2 inhibition downregulated epidermal growth factor receptor variant III (EGFRvIII)-induced lipogenic tumor growth in human glioblastomas [42]. Mutant *KRas* upregulates de novo lipogenic genes, including *FASN*, *ACC1,* and *ACLY*, in lung cancer and gemcitabine-resistant pancreatic cancer cells [43,44], implying that *KRAS*-mutant cancer cells are more sensitive to inhibitors of FA synthesis. Our study showed that EPA treatment inhibited cell visibility and p-STAT3, HPS, and ACC-1, but not FASN expressions in *KRAS*-mutant SUIT-2 cells, suggesting that EPA treatment might reduce ACC-1-mediated de novo lipogenesis to downregulate the tumor growth and survival of pancreatic cancer cells.

Overexpression of *HPS*, a liver-specific gene with hepatocyte mitogenic activity, was previously shown to notably induce lipid accumulation in HepG2 human liver cancer cells through an ERK1/2 pathway [19]. In addition, treatment with the steatogenic reagent, oleic acid, increased HPS expression, whereas the deletion of HPS reduced oleic acid-induced lipid accumulation in HepG2 cells, implying that HPS mediates the regulation of hepatic lipogenesis in liver cancer cells in vitro [19]. Consistent with a previous study, we found that HPS-knockdown reduced SUIT-2 cell viability, and the lipidomics analysis further suggested that the inhibitory effect may be derived from increased EPA expression. Additionally, EPA treatment significantly reduced the cell viability of SUIT-2 pancreatic cancer cells compared to that of hTERT-HPNE normal epithelial pancreas cells, with a dose-dependent decrease in ACC-1 expression. Examination of HPS protein expression in different pancreatic cell lines showed that HPS is highly expressed in most *KRAS*-mutant PDAC cell lines, in which the expression was higher than those in *KRAS* wild-type and/or in normal epithelial pancreas cell lines. In a parallel study, we found that HEK293T cells with constitutively active *RAS* (RAS-V12) showed higher HPS expression upon p-STAT3 activation, suggesting that HPS may be downregulated by STAT3 and may contribute to *KRAS*-mutated pancreatic cancer cell proliferation. It is widely known that *KRAS* is mutated in about 90% of pancreatic cancer cases and contributes to cancer cell proliferation via the MAPK pathway and STAT3 activation; thus, many studies are now investigating ways to target STAT3 as a therapeutic intervention for *KRAS*-mutant pancreatic tumors [13,45]. Taken together, our studies showed that HPS mediates lipid metabolism in human pancreas tumors, which is probably controlled by the *KRAS*/STAT3 signaling pathway, and EPA treatment blocks the KRAS/STAT3 pathway and HPS expression, resulting in the suppression of cell growth and survival of pancreatic cancer cells.

## 5. Conclusions

In the present study, we found that HPS is an important mediator that contributes to lipid metabolism in *KRAS*-mutated pancreatic cancer cells and is involved in cancer cell growth. Furthermore, EPA treatment significantly reduced ACC-1 expression and inhibited STAT3-mediated HPS expression, leading to G_2_/M cell cycle arrest through accumulating cyclin B1 expression. To our knowledge, this is the first study to demonstrate that HPS is mediated by STAT3 and contributes to *KRAS*-mutated pancreatic cancer proliferation. More studies are needed to elucidate the signaling pathway involved in STAT3-induced HPS expression and the HPS-mediated EPA/ACC-1 lipogenic axis in *KRAS-*mutant pancreatic tumors, and our study hereby proposes that HPS is a promising therapeutic strategy for *KRAS*-mutated-driven pancreatic cancer.

## Figures and Tables

**Table 1:** Values of the 50% inhibitory concentration (IC_50_) of omega-3 (n-3) and omega-6 (n-6) PUFA treatments in the SUIT-2 and hTERT-HPNE cell lines.

Times	Cell Lines	n-3 PUFA			n-6 PUFA	
		ALA	EPA	DHA	LA	AA
48 h	SUIT-2	88.5	51.8	140.4	99.8	32.8
	hTERP-HPNE	571.0	1354.0	307.2	230.3	292.0
72 h	SUIT-2	37.2	46.5	51.7	53.7	7.0
	hTERP-HPNE	109.4	160.8	221.9	134.4	122.3

Cell viability data of SUIT-2 and hTERT-HPNE cells with a α-linolenic acid (ALA), eicosapentaenoic acid (EPA), docosahexaenoic acid (DHA), linoleic acid (LA), and arachidonic acid (AA) treatments for 48 and 72 hwere transformed to log(x) and analyzed with log(inhibitor) vs. normalized response using GraphPad Prism 8. IC_50_ data are presented as the half maximal inhibitory concentration (μM).

## Data Availability

The data presented in this study are available on request from the corresponding author. The data are not publicly available due to privacy.

## Supplementary Materials

The following are available online at https://www.mdpi.com/2218-273X/11/3/370/s1, Figure S1. Knockdown of hepassocin (HPS) in SUIT-2 cells changes the lipid profile, Figure S2: Knockdown of hepassocin (HPS) affect cyclins protein expression in SUIT-2 cells.
