# Supplementary material for: Eicosapentaenoic Acid Inhibits KRAS Mutant Pancreatic Cancer Cell Growth by Suppressing Hepassocin Expression and STAT3 Phosphorylation
Source: Biomolecules. 2021 Mar 2;11(3):370. doi: 10.3390/biom11030370 (PMC8001293; doi:10.3390/biom11030370)
Supplement: Supplementary file 1 [file biomolecules-11-00370-s001.pdf]

Supplementary Figure 1.

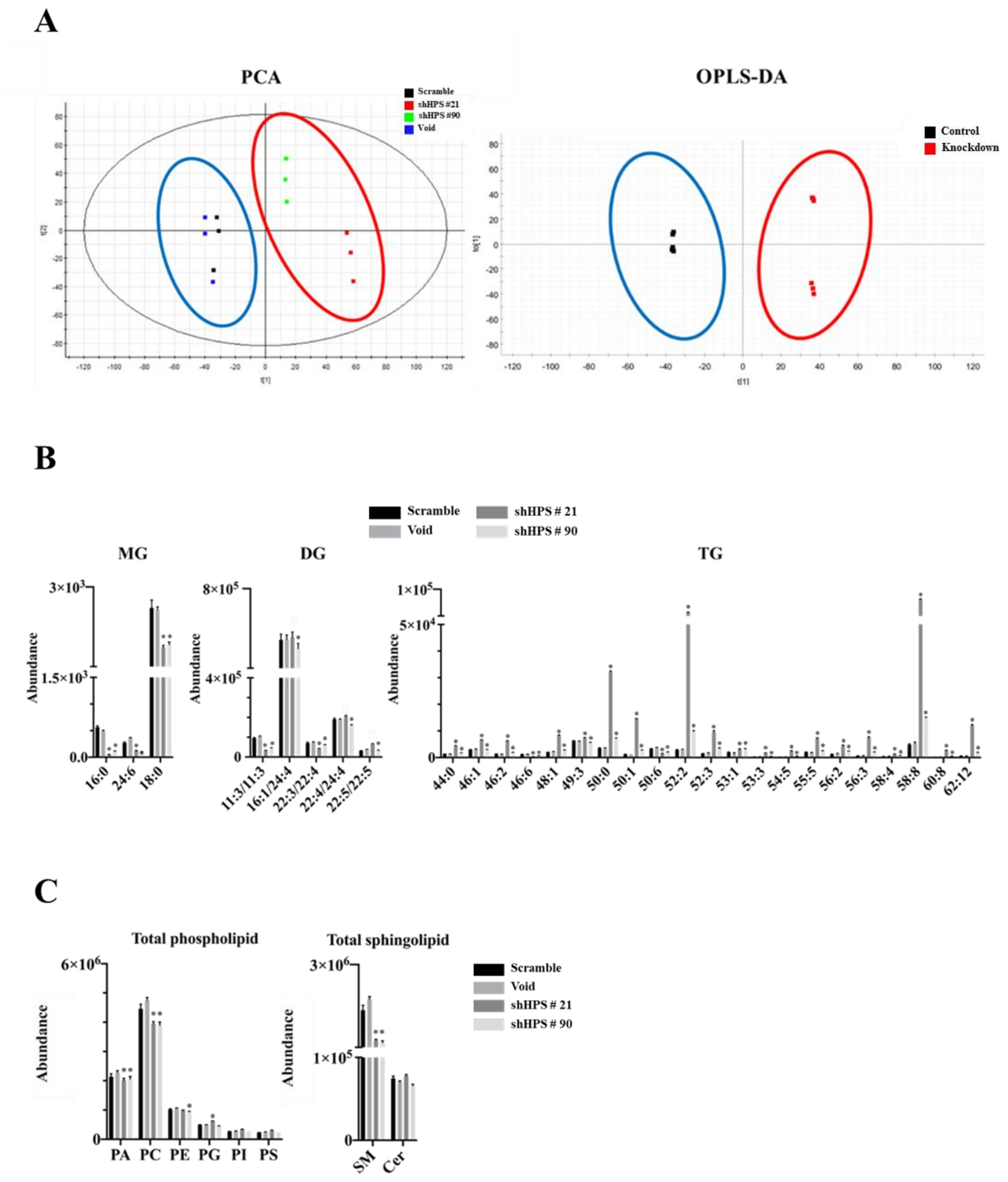

Supplementary Figure 1. Knockdown of hepassocin (HPS) in SUIT-2 cells changes the lipid profile. (A) Left panel, principal component analysis (PCA) and right panel, orthogonal partial least squares discriminant analysis (OPLS-DA) of the lipid profiles of the SUIT-2 cell line with HPS-knockdown and control groups. HPS-knockdown group (short hairpin (sh)HPS#21 and shHPS#90) are shown as red circles, and the control groups (Scramble and Void) are shown as blue circles. (B)

Abundances of monoglycerides (MGs), diglycerides (DGs), triglycerides (TGs), (C) phosphatidic acid (PA), phosphatidylethanolamine (PE), phosphatidylglycerol (PG), phosphatidylinositol (PI), phosphatidylserine (PS), sphingomyelin (SM), and ceramide (Cer) in the indicated SUIT-2 cells with control (Scramble and Void) and HPS-knockdown (shHPS#21 and #90). Results are shown as the mean ± SD of three independent experiment. * $p < 0.05$ vs. the control (by a one-way ANOVA followed by Tukey's post-hoc test).

Supplementary Figure 2.

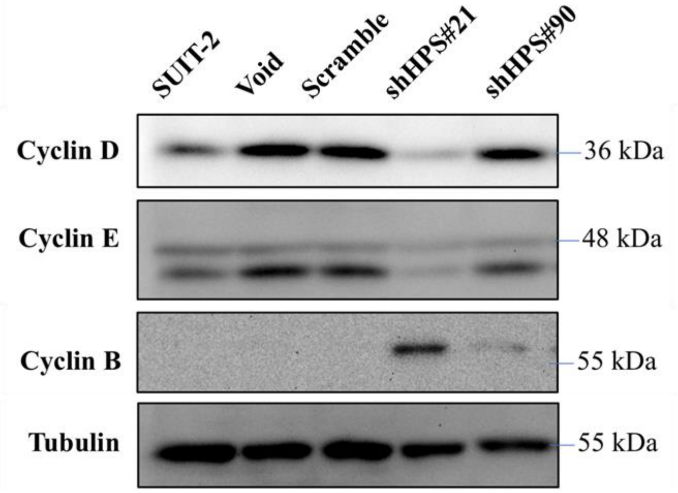

Supplementary Figure 2. Knockdown of hepassocin (HPS) affect cyclins protein expression in SUIT-2 cells. SUIT-2 cells were infected with a lentivirus carrying control vectors of Scramble and Void and the short hairpin (sh) HPS vectors #21 and #90. Cyclin D, E and B expression levels of the indicated SUIT-2 cells were analyzed by Western blotting. Tubulin was used as the internal control.
